# Lipid Profiles Obtained from MALDI Mass Spectrometric Imaging in Liver Cancer Metastasis Model

**DOI:** 10.1155/2022/6007158

**Published:** 2022-10-27

**Authors:** Hee Jung Kwon, Joo Yeon Oh, Kwang Seon Lee, Hyun Kyung Lim, Jisun Lee, Hye-Ran Yoon, Joohee Jung

**Affiliations:** ^1^Department of Pharmacy, Duksung Women's University, Seoul 01369, Republic of Korea; ^2^Duksung Innovative Drug Center, Duksung Women's University, Seoul 01369, Republic of Korea; ^3^ASTA, Inc., Gyeonggi-do 16229, Republic of Korea

## Abstract

Liver cancer metastasis is known to be a poor prognosis and a leading cause of mortality. To overcome low therapeutic efficacy, understanding the physiological properties of liver cancer metastasis is required. However, the metastatic lesion is heterogeneous and complex. We investigate the distribution of lipids using matrix-assisted laser desorption/ionization-mass spectrometry imaging (MALDI-MSI) in an experimental metastasis model. We obtained the differentially expressed mass peaks in comparison between normal sites and metastatic lesions. The relationship of mass to charge ratio (m/z) and intensity were measured, m/z-indicated species were analyzed by MALDI-MS/MS analysis, and identification of these mass species was confirmed using the METASPACEannotation platform and Lipid Maps®. MALDI-MSI at m/z 725.6, 734.6, 735.6, 741.6, 742.6, 744.6, 756.6, and 772.6 showed significantly higher intensity, consistent with the metastatic lesions in hematoxylin-stained tissues. Sphingomyelin SM [d18:0/16:1], phosphatidylcholine (PC) [32:0], PC [31:0], PC [31:1], and PE [36:2] were highly expressed in metastatic lesions. Our results could provide information for understanding metastatic lesions. It suggests that the found lipids could be a biomarker for the diagnosis of metastatic lesions.

## 1. Introduction

Liver cancer is common and is a leading cause of mortality worldwide [1] and in South Korea [2]. Liver cancer metastasis is one of the causes of death in patients with liver cancer and shows in the lungs, portal vein, and portal lymph nodes [3]. To develop a strategy for liver cancer treatment, an understanding of the liver cancer progression is required. However, heterogeneous cancer is complex. In addition, the microenvironment of liver cancer plays a key role in cancer progression [4]. To explicate the properties of liver cancer, histological, and cytopathological investigation were performed using biomarkers by specialists. Thus, many researchers investigate to figure out the biomarker and elucidate the relationship of the biomarker to liver cancer progression. Nevertheless, it is an unmet need in the liver cancer field.

Various analytical methods have been applied to cancer research. One of them, matrix-assisted laser desorption/ionization (MALDI)-mass spectrometric imaging (MSI) is a powerful technique for showing molecular distributions in tissue sections. MALDI-MSI reveals the profiles of peptides, lipids, or metabolites onto tissue slides [5]. A recent report suggests that this method could analyze the chemical communication between tissues and cells [6]. Especially, lipid profiles using MALDI-MSI were investigated in colorectal cancer metastasis [7] and metastatic breast cancer [8] as well as primary cancers. However, the lipid distribution in liver cancer metastasis was not reported. In this study, we observed the lipid profiles in the liver cancer metastatic model using MALDI-MSI.

## 2. Materials and Methods

### 2.1. Cell Cultures

SK-Hep1_Luc cells were maintained in Dulbecco's Modified Eagle Medium (Thermo Fisher, MA, USA) including 10% fetal bovine serum (GW Vitek, Seoul, Korea) and 1% penicillin-streptomycin (GenDEPOT, TX, USA). The cells were incubated in a 5% CO_2_ incubator at 37°C.

### 2.2. Liver Cancer Metastasis Animal Model

The animal experiment was performed following the protocol approved by the Institutional Animal Care and Use Committee of Duksung Women's University (2019-003-009). Extremely immune-deficient (NRGA) mice (male, 5-week-old) were purchased from JA BIO (Suwon, Korea). Mice were acclimatized for 1 week before the experiment. One million SK-Hep1_Luc cells were intravenously injected into the mice [9]. For monitoring, D-luciferin (PerkinElmer, EU) was intraperitoneally injected into the metastasis animal model and the luminescence was detected by an *in vivo* imaging system (VISQUE in vivo Elite, Vieworks, Gyeonggi-do, Korea) [10].

### 2.3. Tissue Preparation

At 30 days after transplantation, mouse liver tissues were isolated. The tissues embedded into the OCT compound (Leica FCS22, Nussloch, Germany) were cut into a 10 *μ*m-thick section using a cryotome (Leica CM1520, Leica). Tissue sections were applied to indium tin oxide (ITO) coated slides. For the histological analysis of tissue sections, tissue sections were stained with hematoxylin (H staining) and observed using a microscope (Leica).

### 2.4. MALDI-MSI Analysis

Tissue sections were washed with ethanol and dried in a vacuum desiccator. The MALDI matrix at 1.1 mL DHB was used per tissue sample using a spraying device (ImagePrep, Bruker Daltonics, Inc., Bremen, Germany). MALDI-MSI was acquired with MALDI-time of flight (TOF)-based mass spectrometer (IDSys Premier, ASTA Inc., Korea) [11] detecting positive ions in a mass range between m/z 600 and m/z 1,500 with 50 *μ*m of the spatial solution, 50 shots per pixel, and 1 kHz laser frequency. MSIviewer (ASTA Inc.) was used to acquire mass imaging and spectra of the region of interest. Identification of ions in specific lesions was analyzed in MS/MS and searched by the MassBank of North America database (MoNa, https://mona.firehnlab.ucdavis.edu), Lipid Maps® (https://lipidmaps.org), and METASPACE annotation platform (https://metaspace2020.eu).

### 2.5. Statistics

The intensity of metastasis and normal tissues was compared and represented as mean ± standard deviation. Statistical significance was calculated by Student's *t*-test. A probability *p* value of <0.005 was considered to indicate statistical significance.

## 3. Results and Discussion

### 3.1. Imaging of Liver Cancer Metastasis

**Figure 1:**
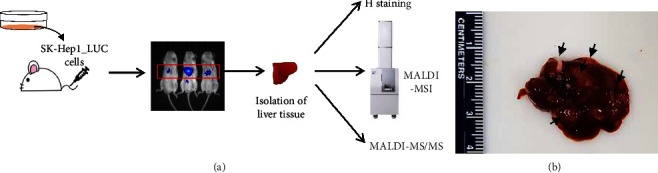
Analysis procedure. (a) Scheme of metastasis model and imaging analysis. (b) Liver cancer metastasis in mouse liver tissues (black arrow, liver cancer metastasis).

To observe lipid profiles in metastasis, the liver cancer metastasis was produced by the experimental metastasis models. We monitored metastasis using the *in vivo* imaging system. Human liver cancer SK-Hep1 cells got mainly settled in mouse liver (Figure 1(a), red box). The liver cancer metastasis could be observed in the isolated mouse liver tissues (Figure 1(b), black arrows). Our result of the experimental metastasis model mimicked how liver cancer cells induce metastasis in patients. Metastatic lesions in our model were similar to sites of metastasis in liver cancer patients [3].

### 3.2. Lipid Profiles of Metastatic Lesions and Normal Tissues

**Figure 2:**
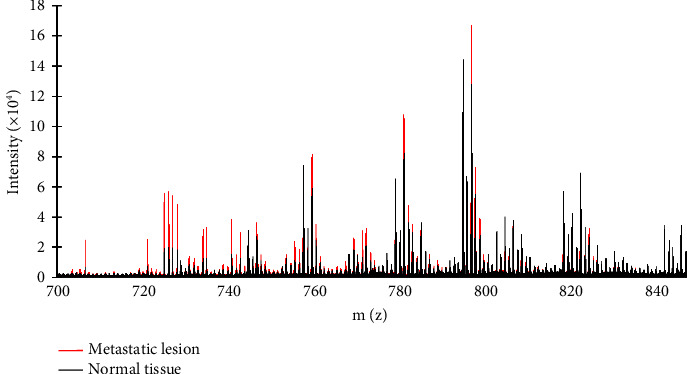
Comparison of intensity between the metastatic lesion and normal tissues. The spectrum of molecular species in the m/z 700～850 range. Four slides of each sample were determined by MALDI-MS. Five spots per slide were selected.

The spectrum of MALDI-MS analysis in liver tissues obtained from the liver cancer metastasis model is shown in Figure 2. Intensity (*y*-axis) represented the average in each m/z value. Metastatic lesions showed higher intensity than normal tissues between m/z 700 and m/z 800. The range of molecular weight from 700 to 800 is composed of sphingomyelin (SM) and phosphatidylcholine (PC) [12]. Our results also demonstrated that common daughter ion m/z 184 (phosphocholine as the fragment of SM and PC) was detected by MALDI-MSI and UHPLC-MS/MS (Supplementary Figure 1). To select and identify m/z species, the ratio of the metastatic lesions and normal tissues (M/N ratio) was calculated. The M/N ratio was summarized in Table 1. The lists showed that the M/N ratio is over 2.5 or below 0.5 with *p* < 0.005. To identify these mass species, open access database (MoNa, Lipid Maps®, and Metaspace annotation platform) was utilized. The expected formulas of these masses are shown in Table 1.

The spectrum of molecular species is in the m/z 700∼850 range. Four slides of each sample were determined by MALDI-MS. Five spots per slide were selected.

**Figure 3:**
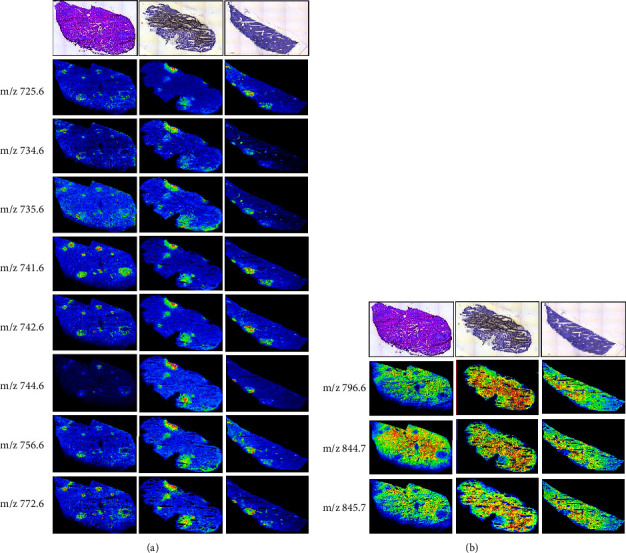
MALDI-MSI of metastatic lesions. (a) Upexpression of m/z species and (b) downexpression of m/z species in liver cancer metastatic lesions. The top panel is the hematoxylin-stained image. MALDI-MSI is the image showing the significant difference between the metastatic lesions and normal tissues at the specific mass.

Eight m/z showed higher intensity in metastatic lesions than in normal tissues and three m/z decreased intensity. As shown in Figure 3, MALDI-MSI was correlated with H-stained histological images. Selected mass species showed remarkable differences in MALDI-MSI (Figure 3(a)). Red or yellow spots represented high intensity coincided with the metastatic lesions observed in the H-stained tissue section. On the other hand, m/z 796.6, 844.7, and 845.7 showed a small ratio (M/N). Blue spots were observed in the metastatic lesions (Figure 3(b)).

### 3.3. Sphingomyelins and Phosphatidylcholines as Biomarkers of Liver Cancer Metastasis

The large M/N ratio was selected and analyzed to identify these species using MALDI-MS/MS (Table 2). Analyzed MS/MS data were compared with mass spectrum in the MoNA database, Lipid Maps®, and METASPACE annotation platform. We figured out five expected lipids of mass species: SM [d18:0/16:1], PC [32:0], PC [31:1], PC [31:0], and PE [36:2]. Interestingly, SM[d18:0/16:1] and PC [32:0] were observed in the formula of sodium salt (Na^+^) and potassium salt (K^+^).

Sphingolipids control the signaling for fundamental cellular processes in various cancer [13]. Sphingosine as a backbone of sphingolipids is metabolized to ceramide, sphingosine-1-phosphate (S1P), SM, and so on [14]. Especially, the balance of S1P and C18-ceramide regulates cancer cell survival [15]. SM is well known to inhibit apoptosis and induce proliferation [16]. Thus, SM plays a key role in cancer progression [17]. Our results indicated that SM [d18:1/16:0] was remarkable in liver cancer metastatic lesions. The high intensity of m/z 741.6 (SM [d18:1/16:0]) also shows in thyroid papillary cancer [18], HER2-positive breast cancer [19], and gastric cancer [20]. However, the expression of SM [d18:1/16:0] is low in prostate cancer [21]. In addition, the high intensity of m/z 725.6 (SM) shows in lung cancer [22].

PC and its metabolites regulate proliferation, survival, migration, and drug resistance [23]. Thus, the regulation of phospholipid metabolism is a good target for cancer treatment [24]. Six breast cancer cell lines show different levels of PCs. PC [32:0] is commonly observed in the breast, lung, colorectal, esophageal, gastric, and thyroid cancer [25]. Lung adenocarcinoma A549 cells and PC9 cells also show the expression of PC[32:0] [26].

The mainly observed two lipids in liver cancer metastasis, SM [d18:1/16:0] and PC [32:0] indicate different aspects in several cancers compared with normal cells. These lipids are considered to contribute to liver cancer progression. In this study, our results firstly reveal that these lipid molecules are significantly expressed in liver cancer metastasis.

## 4. Conclusions

Some m/z showed higher intensity in metastatic lesions than normal tissues from MALDI-MSI analysis. These results suggest that MALDI-MSI could diagnose liver cancer metastasis. Among several species identified from MALDI-MS/MS, cations of SM [d18:0/16:1] and PC [32:0] were mainly expressed. There are expected to be biomarkers for liver cancer metastasis.

## Figures and Tables

**Table 1:** Intensity of mass to charge ratio (m/z) in the metastatic lesion and normal tissues.

m/z	Metastatic lesion (M)	Normal tissues (N)	Ratio (M/N)	Expected formulas
725.6	14234 ± 2973	4200 ± 185	3.38	[C_39_H_79_N_2_O_6_P+Na]^+^
734.6	18975 ± 2609	5578 ± 526	3.4	[C_40_H_80_NO_8_P+H]^+^
735.6	10289 ± 1033	4034 ± 165	2.55	[C_39_H_76_NO_8_P+NH_4_]^+^
741.6	24854 ± 4407	7160 ± 287	3.47	[C_39_H_79_N_2_O_6_P+K]^+^
742.6	11668 ± 2129	4149 ± 226	2.8	[C_39_H_78_NO_8_P+Na]^+^
744.6	11416 ± 1996	4310 ± 78	2.6	[C_41_H_78_NO_8_P+H]^+^
756.6	32132 ± 5905	12528 ± 75	2.56	[C_40_H_80_NO_8_P+Na]^+^
772.6	50062 ± 6438	16650 ± 1088	3	[C_40_H_80_NO_8_P+K]^+^
796.9	3876 ± 266	9240 ± 610	0.42	[C_42_H_80_NO_8_P+K]^+^
844.7	7583 ± 2404	18497 ± 230	0.4	[C_48_H_95_NO_9_P+H]^+^
845.7	5193 ± 1094	10668 ± 52	0.48	[C_46_H_82_NO_8_P+K]^+^

**Table 2:** Identification of mass obtained from liver cancer metastatic lesions.

Common names (abbreviation)	Mass (m/z)
SM [d18:0/16:1]	725.6, 741.6
PC [32:0]	734.6, 756.6, 772.6
PC [31:1]	735.6
PC [31:0]	742.6
PE [36:2]	744.6

SM: sphingomyelin, PC: phosphatidylcholine, and PE: phosphatidylethanolamine.

## Data Availability

The data used to support the findings of this study are included within this article and the supplementary information files.

## Supplementary Materials

Supplementary Materials Figure S1: UHPLC-MS/MS and MALDI-MSI analysis data.
