# Supplementary material for: Lipid Profiles Obtained from MALDI Mass Spectrometric Imaging in Liver Cancer Metastasis Model
Source: Int J Anal Chem. 2022 Oct 27;2022:6007158. doi: 10.1155/2022/6007158 (PMC9633205; doi:10.1155/2022/6007158)
Supplement: Supplementary Materials — Figure S1: UHPLC-MS/MS and MALDI-MSI analysis data. [file 6007158.f1.pdf]

A **B**

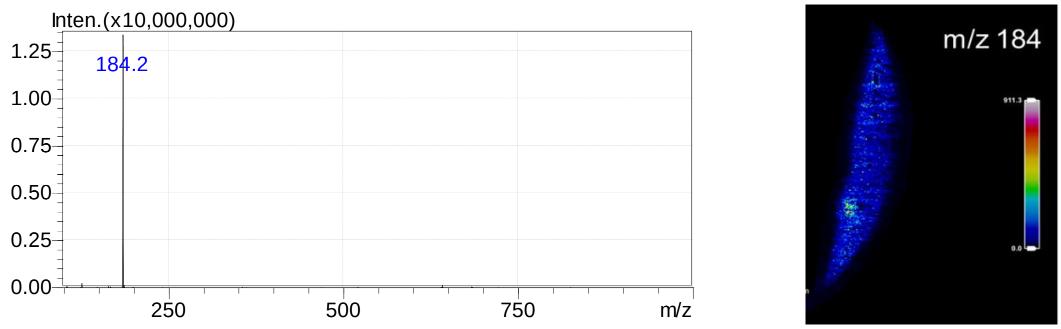

Supplementary Figure 1. Spectrum and MALDI-MSI of daughter ion m/z 184

(A) Spectrum of liver tissue including metastasis by UHPLC-MS/MS

(B) MALDI-MSI of liver tissue including metastasis

Supplementary Data

Ultra-performance liquid chromatography and MS/MS analysis

The UHPLC-MS/MS system comprised a Shimadzu UHPLC SCL-40 system hyphenated to a triple quadrupole mass spectrometer LCMS-8060NX (Shimadzu Corporation, Kyoto, Japan). The samples were kept in the autosampler (Nexera X2 SIL-30 AC) at 4°C. The Chromatographic separations were performed using a 150 mm × 2.1mm, 1.7 µm Acquity UPLC BEH C8 column (Waters, Milford, MA, USA). The LC system was composed of one Nexera LC-40D XS quaternary pump, a column oven (CTO-40C) maintained at 40°C (±1°C), and a model DGU-405 degasser. The separation was performed using gradient flow of a solvent; mobile phase A consisted of 10% methanol (containing 5 mM ammonium acetate) and mobile phase B consist of 20% isopropyl alcohol, 30% acetonitrile, and 50% methanol. Gradient conditions were as follows: Starting at 45% mobile phase B (hold time 1.5 min) mobile phase B was increased linearly to 100% in 10 min remaining at 100% mobile phase B until 16min and re-equilibrated at initial conditions at 4 min. The total run time was 20 min. The flow rate was set at 0.3 mL/min and delivered to the MS. The injection volume was set at 1µL. Nitrogen gas (99.99 %) was supplied by an ATN-1050 Nitrogen gas generator (Shimadzu, Kyoto, Japan).

Sample analysis using production mode was performed in positive ion mode in the range of m/z 150-1000. Instrument setting; Collision energy, -35 eV; Source temperature, 100°C; Desolvation Temperature, 650°C; Dissolution line (DL) temperature, 250°C; Heat block temperature, 400°C; Nebulizing gas flow, 3 L/min; Drying gas flow was set at 10 L/min. Data was collected and analyzed by Lab Solution (Shimadzu, Japan).
